# Koch on Disinfectants

**Published:** 1882-11

**Authors:** 


### Koch on Disinfectants.

From careful investigations, Koch concludes that the only certain disinfectants are chlorine, bromine and corrosive sublimate, and that to arrest development, only corrosive sublimate, certain ethereal oils, thymol and allyl-alcohol are available. Bromine vapors are recommended for confined spaces. Chlorine is a little less satisfactory, but more so than formerly supposed. In all cases where neither heat nor gases are available, corrosive sublimate, and indeed all the mercurial salts are recommended.

A solution of 1 per 1,000 of the mercuric chloride, sulphate or nitrate, killed the resting spores in ten minutes; and indeed simple moistening of the earth containing the spores with this solution is sufficient to arrest their power of development. Solutions of 1 in 1,000 to 1 in 15,000 are sufficient to kill micro-organisms. The poisonous action of such diluted solutions may be disregarded. The cost also is far below that of carbolic acid.—*London Medical Record*, Aug. 15th.

